# Digital Health Solutions for Mental Health Disorders During COVID-19

**DOI:** 10.3389/fpsyt.2020.582007

**Published:** 2020-09-09

**Authors:** Alton Ming Kai Chew, Ryan Ong, Hsien-Hsien Lei, Mallika Rajendram, Grisan K V, Swapna K. Verma, Daniel Shuen Sheng Fung, Joseph Jern-yi Leong, Dinesh Visva Gunasekeran

**Affiliations:** ^1^National University of Singapore (NUS), Singapore, Singapore; ^2^UCL Medical School, University College London (UCL), London, United Kingdom; ^3^School of Medicine and Medicine Science, University College Dublin (UCD), Dublin, Ireland; ^4^NUS Saw Swee Hock School of Public Health (NUS-SSHSPH), Singapore, Singapore; ^5^Institute of Mental Health (IMH), Singapore, Singapore; ^6^Duke-NUS Medical School, Singapore, Singapore; ^7^Raffles Medical Group, Singapore, Singapore

**Keywords:** coronavirus disease 2019 (COVID-19), global mental health, psychiatry, digital health, pandemic

## Introduction

The Coronavirus disease 2019 (COVID-19) pandemic has had an immense impact infecting 10 million individuals and claiming 500,000 lives globally as of 1 July 2020 (1). The rapid spread was largely enabled by the onset of the outbreak in Wuhan city just prior to the Lunar New Year season, a peak period in travel to and from China (2). Fortunately, many regions have controlled initial outbreaks and shared their experiences. These have been recently summarized by the World Health Organization (WHO), highlighting the importance of developing targeted responses and enhancing communication to address the pandemic’s impact (3, 4).

Notably, emotionally driven sharing of misinformation has featured prominently in this crisis, fueling both confusion and irrational anxiety among the public (5, 6). Termed an “infodemic”, this has far-reaching consequences on population health with a direct impact on overloaded health systems and an indirect impact on mental health, resulting in paranoia and behavioral responses like stock-piling due to disproportionate fear (7). The impact of misinformation in the media on public emotion and fear has been illustrated with the Middle-East Respiratory Syndrome (MERS), whereby it led to a surge in fear and sustained economic consequences (8).

### Mental Health and COVID-19

The psychosocial impact of large-scale disasters and previous outbreaks have been described, including increased incidence of mental health disorders (9). Similarly, COVID-19 has had a two-fold detrimental impact on the mental health of populations subject to the psychosocial consequences of the pandemic, including the incidence of new onset mental health disorders as well as deterioration in the condition of patients with existing mental health disorders (4, 10). This impact is on the rise given the protracted lock downs, social isolation, and concomitant occupational stressors in the context of the weakened global economy (10–12). These factors highlight the urgent need to scale-up and decentralize mental health services to attain a multiplier effect in the provision and accessibility of these services to combat the pandemic-driven surge in mental health disorders (9, 11).

Fortunately, several reports have demonstrated the effectiveness of digital health solutions for various applications, including addressing gaps in mental health services (12). These solutions include cloud-based big data systems, artificial intelligence (AI)–based chatbots, online health communities (OHCs), and telehealth platforms. Several have already been extensively applied for the pandemic’s direct impact on health, such as big data systems and telehealth for remote consultations (13, 14). This review summarizes relevant applications of digital health that can help address the indirect impact of the pandemic on population mental health.

### Cloud-Based Big Data Systems

Cloud-based big data systems have been successfully applied in previous infectious disease outbreaks by aggregating data from numerous possible sources including weather surveillance systems (15), queries in online search engines (16), and even connected devices among the Internet of Things (IoT) such as mobile phones and drones (17, 18). Applications of these systems range from early detection of outbreaks to facilitating global digital epidemiology collaborations that address unresolved clinical uncertainties, such as ocular findings for early detection of latent tuberculosis (19, 20). Successful applications include monitoring dengue outbreaks using data on mobility from mobile phones (15) or queries in search engines such as Baidu in China (21).

Evidence is emerging for the value of these platforms beyond retrospective or real-time surveillance applications, to prospective projections of disease trends and clinical need. In the context of the ongoing pandemic, several potential applications of these tools have emerged, such as predicting outbreaks of COVID-19 based on historic travel data and public health capacity (22). Also, Cornelia Betsch and the COVID-19 Snapshot MOnitoring (COSMO) group evaluated methods for surveillance of behavioral responses to the pandemic (5). These applications enable evidence-based approaches to localize public health responses and monitor their effectiveness, in accordance with WHO recommendations (23).

Related applications for mental health include the prediction of disorders such as depression, stress and anxiety, using publicly available data from websites like Twitter (24). These applications are gaining traction in academic consciousness as digital data becomes more ubiquitous, as exemplified by the development of recommendations for evidence-based research using tools like Google search to predict mental disorders (24, 25). There are also validated individual-level applications of big data, such as the use of ecological momentary assessment (EMA) from passive behavioral monitoring of mobile data, that have been used to detect and monitor severity for a spectrum of mood and behavioral disorders (26). This ushers in the possibility of precision digital mental health with tailored recommendations to the individual, as recently described for panic disorder (27).

These methods can be leveraged for useful applications during lockdowns, such as early detection of mental health disease onset or progression. However, unresolved barriers to implementation include ethical and privacy issues of population-level monitoring such as with big data systems for contact tracing, that would similarly apply to systems for mental health surveillance (28). Measures to facilitate implementation include the use of high quality input data and clinical validation using formal diagnostic criteria, robust methodology, and actionable outcomes (29). Nonetheless, these systems can contribute to responses to the pandemic and address the needs of the vulnerable groups during the recurrent lockdowns in response to local outbreaks, such as potential victims of domestic violence (9).

### AI Chatbots

AI chatbots utilize pre-programmed content and decision-trees for automated conversations using techniques such as natural language processing (NLP). These are more interactive than static digital repositories leading to higher engagement for patients (30). Preliminary reports of AI chatbots that have been developed for mental health include solutions providing counseling for well individuals to improve psychological well-being (31). Others include AI chatbots such as Wysa for digital mental well-being with demonstrated effectiveness in patients with depression (32), and Woebot for cognitive behavioral therapy (CBT) in young adults with depression/anxiety symptoms (33).

These tools have potential applications in the current pandemic and beyond for preventive care and mental health promotion. They also function as contingency solutions to expand surge capacity in the event of overwhelming clinical need (30). However, their applications needs to be supervised given limited clinical validation with robust experimental design (34). Other challenges clinical AI have also been described in various specialities, including practical, technical, and sociocultural barriers to implementation (35, 36). Particularly given the conversational nature of chatbots and linguistic variations in different populations, acculturation is needed to facilitate the implementation of chatbots in new populations, as demonstrated with AI chatbots for health professional training to address colloquialisms such as “Singlish” in Singapore (37). This is crucial to ensure emotional support or triage advice are perceived accurately by patients, and piloting messages will help ascertain effectiveness (38).

Validated community mental health assessment tools could be incorporated in future conversational AI chatbots to prompt regular self-reporting by patients of wellness and social inclusion for active population monitoring. These include the various iterations of the Social and Communities Opportunities Profile (SCOPE) scale validated in the United Kingdom and Hong Kong, as well as the mini-SCOPE in Singapore (39). Applying AI chatbots in this manner using a “sorting conveyor” operational model could be transformative, whereby the AI solutions built with predefined criteria can re-direct individuals requiring more comprehensive psychological support to appropriate services within a stepped-care mental health service (9).

### Online Health Communities

Open digital patient engagement platforms that allow any visitor to a website or application to view interactions between patients and/or healthcare providers are called online health communities (OHCs). OHCs could be the silver bullet to the “infodemic”, which is largely attributed to the unfettered spread of viral misinformation in unverified sources or platforms like social media, crowding out official communication (12, 40). In the earlier example of the impact of misinformation on fear during MERS, Choi et al. found that it created a positive feedback loop leading to a spiral of growing misinformation and paranoia, with the publication of more inaccurate information by the media in a bid to capitalize on public interest (8).

**Figure 1:**
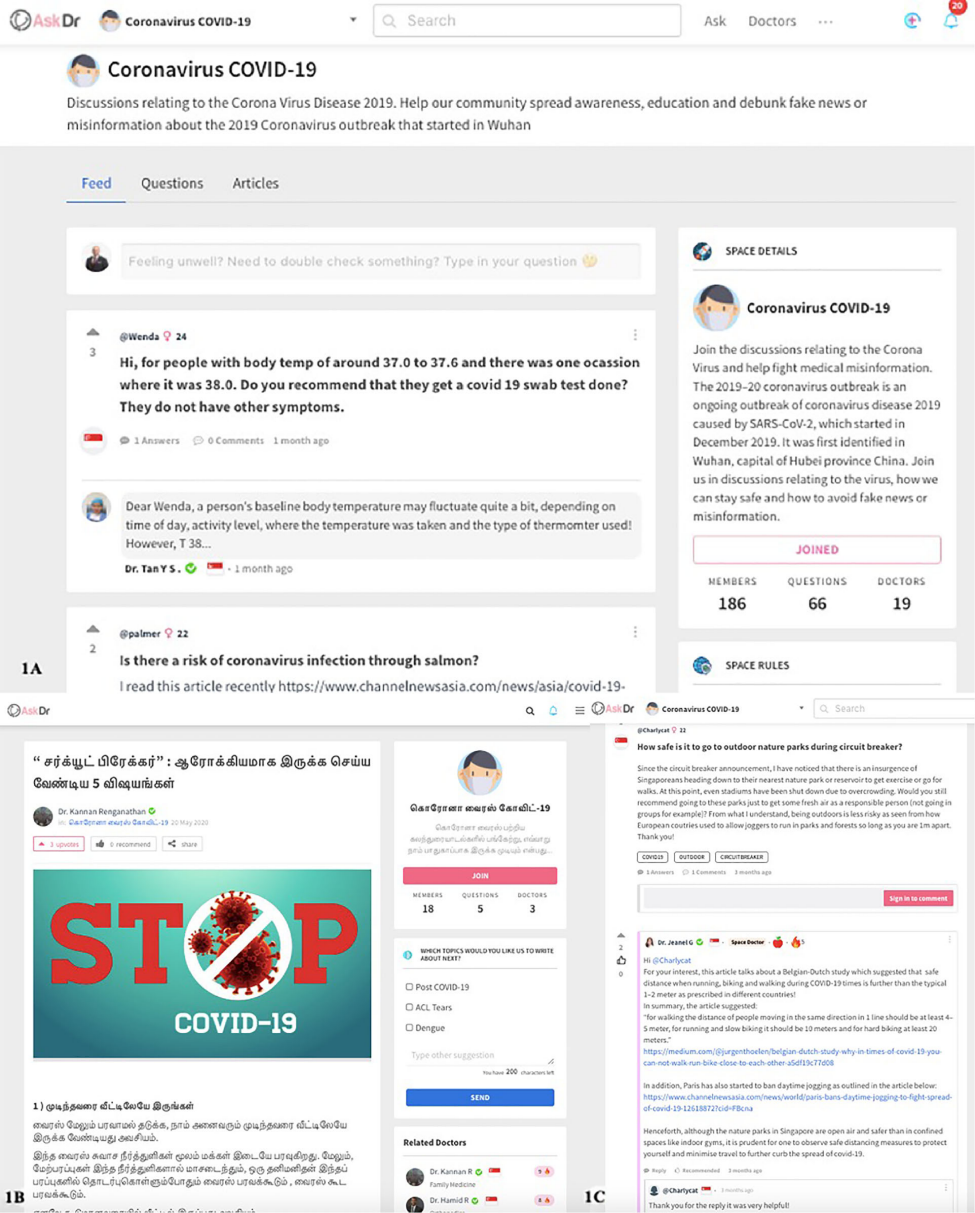
The ideation of a free, crowd-sourced digital tool, and **(A)** online health community (OHC) moderated by verified providers incorporating **(B)** multilingual patient education content and **(C)** personalized health promotion based on demographic information to address the needs of patients with varied backgrounds. This enables sustainable public health promotion for patients and gamified digital tools for providers to address medical misinformation, project their expertise online, and engage members of public with relevant interests.

Big data systems such as the aforementioned COSMO for behavioral surveillance provide measures of these phenomena to develop targeted public health communication messages—an essential first step to combat this problem (38). However, due to the speed of misinformation propagated online, there is increasingly a need to implement a digital effector arm for our monitoring systems (3), one that amplifies reputable sources to directly combat misinformation in a transparent, scalable manner by addressing myths and promoting reputable sources of information (41). In Singapore, such a solution was developed by AskDr through needs-finding surveys and ideation with frontline providers (**Figure 1**). It combines network effects of social media with behavioral gamification to give registered medical professionals digital tools to crowd-source a coherent counter-narrative to misinformation (42).

Public health agencies should similarly develop or adopt such tools for the “last mile” of public health communication. In the context of the ongoing pandemic, key applications include promoting reliable information and directly breaking the “spiral of misinformation”. Direct potential applications of OHCs for patients at-risk of mental health disorders include lowering the barrier to access care and support for stigmatized illnesses such as anxiety and depression, by allowing patients to seek initial medical advice anonymously (43). Apart from the provision of basic demographic information such as gender and age that are required to contextualize medical advice; otherwise, anonymous engagement also helps to address limitations such as privacy issues similar to those with big data systems (28).

Other applications of OHCs that can enhance public health responses to the pandemic include provision of triage advice to optimize right-siting of patients and reduce unnecessary healthcare presentations where appropriate. This “tele-support” can be used long-term for fundamental illness-related concerns that may not require formal consultation, such as questions about potential interactions of chronic medications with over-the-counter (OTC) medications or other health products (44). Finally, they provide an avenue for asynchronous patient engagement between outpatient appointments while protecting the privacy of healthcare providers, creating opportunities for patient support and early identification of at-risk individuals needing to be re-directed to formal mental health services online or in-person (9).

### Telehealth Platforms for Remote Consultation

Digital telehealth services have numerous embodiments including video-conferencing, store-and-forward technology, remote tele-monitoring with connected devices, and mobile health applications, all of which are increasingly applied in large-scale disasters (45). These can be used for either Asynchronous or Synchronous consultations with private discussions between patients and healthcare providers (46). Existing descriptions of tele-mental health services indicate the importance of human support and interaction regardless of the embodiment of telehealth used (6, 12). Although its application in COVID-19 for mental health services has been greatly enabled by legislative changes (6), the barriers to telehealth adoption that have kept it from becoming mainstream to date still remain (47). Ensuring successful, sustained adoption requires active alignment with clinical needs when deploying services (6).

Nonetheless, tele-mental health services are critical to maintain the continuity of care for patients with mental health disorders by providing avenues for remote review and prescription re-fills (9). Other avenues with long-term value to health systems include co-ordinated avenues for health professionals to engage patients with mental health disorders more frequently, facilitate early detection of those at-risk of self-harm, and enable preventive interventions such as motivational interviewing that reduce hospitalizations (11, 48, 49). Apart from the traditional two-way teleconsultation between doctor and patient, multi-way conferencing or tele-collaboration by allied professionals remotely supported by clinicians has been described (50) and is mainstreamed in countries like Singapore to project tertiary care to nursing homes and intermediate and long-term care (ILTC) facilities.

## Discussion

COVID-19 is the first “viral” pandemic that threatens to overwhelm mental health services in coming months as a result of fear perpetuated by misinformation alongside social isolation during lockdowns (4, 11) These unprecedented challenges highlight the need to develop creative solutions to address the impending surge in mental health disorders (4, 10). The four technologies discussed in this review are potential avenues to expand the capacity and penetration of existing mental health services to address this indirect health impact of the pandemic. Hybrid strategies combing various solutions in an overarching “pyramid” operational model may be required to rapidly scale-up stepped mental health services.

This was illustrated in the SAVED study operationalizing telehealth for complexed emergency services (51). Digital operationalization of mental health services can be similarly achieved using combinations of digital tools in comprehensive services such as Illness Management and Recovery (IMR) Programs (52). IMRs are structured mental health services incorporating multi-modal mental health interventions to promote self-management and optimize treatment. Pioneered in America, they were externally validated and demonstrated to reduce re-admissions and the post-illness recovery period of Asian patients after discharge from in-patient psychiatric services (52).

The pyramid base catering to the needs of the general population could include screening tools such as big data systems and/or OHCs to actively identify and/or engage at-risk individuals without pre-existing mental health disorders, as well as provide tele-support services to reduce risk of progression in patients with mental health disorders (49). As countries re-open, at-risk individuals can be directed to AI-based chatbots providing automated support as well as triage in a “sorting conveyor” operational model to further escalate care as appropriate to in-person or telehealth mental health services based on patient risk profile (3, 16). These requires modifications to traditional practice as described for telehealth cognitive processing therapy (CPT) services to treat post-traumatic stress disorder (PTSD), a condition likely to increase in coming months even among healthcare professionals due to the prolonged stress of frontline services or rationalizing care in some regions (4, 53).

### The Way Forward: Intentional Deployment of Digital Mental Health

Ultimately, the effective deployment of digital mental health services is greatly dependant on successful assimilation within existing health systems. Patient willingness to use, provider acceptance, and even the quality of digital and hardware infrastructure are fundamental considerations that need to be addressed. This has been recently illustrated based on the challenges of implementing AI solutions for Ophthalmology despite maturity of the technology (35, 36). Deployment of digital health thereby needs to be driven by the needs of the target patient population, clinical acceptance, and validated effective applications (38). These considerations dictate the likely effective form of deployment for these digital tools.

Designing effective digital mental health care requires taking into account the wide range of patient needs determined by the severity of mental health disorder(s), social determinants of health (SDH), access to technology, and cultural acceptance, among others (35, 38). There is no “one size fits all” solution, and research in telehealth has demonstrated that individualized design considerations are critical to maximize acceptance, ensure effectiveness, and sustain adoption with recurrent use (38). Meeting the needs of patients in a timely and cost-effective manner ensures sustained adoption beyond the COVID-19 crisis. For provider adoption, stakeholder engagement methods have been advocated to map out clinical processes, participants, and individual responsibilities to actively plan deployment for telehealth (6) and are just as important for other forms of digital health (47).

Firstly, this requires detailed mapping of the needs, roles, and incentives of stakeholders such as healthcare workers, logistic procurement teams, and chief medical informatics officers. They are prioritized into primary and secondary stakeholders based on their capacity to make or influence decisions about adoption of digital tools. Subsequently, a deployment strategy is developed to maximize stakeholder alignment while minimizing disruption to existing processes or new responsibilities that may overburden stakeholders. This also yields crucial insights for communication strategies to engage individual stakeholder groups effectively.

Participatory approaches like these with design-thinking have been used to operationalize tele-health in complexed emergency services (51), as well as develop solutions with targeted applications such as AI chatbots for automated adolescent mental health coaching (54). In tandem, it is important to address the needs of vulnerable populations that may fail to seek care, such as potential domestic violence or child abuse victims (9). They may require tailored solutions such as targeted deployment of mobile mental health services provided by allied mental health professionals that could be remotely advised by psychiatrists using “hub-and-spoke” telehealth to project services into these pockets of society.

## Conclusions

In conclusion, the massive health impact of the first “viral” pandemic has been fueled by global travel, social isolation, rampant misinformation in social media, and other intricacies of modern life. However, digital mental health tools are the silver lining we are fortunate to have, as they can empower responses to the COVID-19 outbreak at a scale that was never before possible in human history. Responding effectively to the mounting impact of this pandemic on population mental health may ultimately require us to leverage these digital health solutions to expand the capacity of mental health services and supplement face-to-face care with an intentional approach for successful deployment (6, 47).

## Author's Note

Authors AC and RO are medical students on clinical research attachment with author DG. Author H-HL is an adjunct Associate Professor at the Saw Swee Hock School of Public Health (SSHSPH), NUS, and concurrently Chief Executive Officer, The American Chamber of Commerce in Singapore. Author RM is a Tutor in Academic English at the Center for English Language Communication (CELC), NUS. Author GK is a Senior Operational Manager at the Institute of Mental Health (IMH), Singapore. Authors SV, DF, and JJ-YL are Senior Consultant Psychiatrists at IMH, Singapore. Author SV is also a Professor at Duke-NUS, Singapore. The author DF is also the Chairman Medical Board at IMH, Singapore, as well as President of the International Association of Child and Adolescent Psychiatry. DF is concurrently adjunct Associate Professor at all three medical schools in Singapore, NUS, Duke-NUS, and LKC. DG is a Senior Lecturer and Faculty Advisor (Medical Innovation) at the National University of Singapore (NUS), and Physician Leader (Telemedicine) at Raffles Medical Group.

## Author Contributions

Authors AC, RO, and DG conceptualized the manuscript, researched its contents, wrote the manuscript, and edited all revisions. Authors H-HL, RM, GK, SV, DF, and JJ-YL intellectually contributed to the development and writing of the manuscript, added text, and edited all revisions.

## Conflict of Interest

DG reports equity investment in Doctorbell (acquired by MaNaDr, Mobile Health), AskDr, VISRE, and Shyfts, and appointment as Physician Leader (Telemedicine) at Raffles Medical Group. He reports advisory to university-affiliated technology developers and start-up companies developing patient engagement systems in Southeast Asia. He declares receipt of travel funding from the Commonwealth Fellowship in Innovation award, Mobile Health Education grant, and National Youth Fund award, for clinical research training and collaborations at Oxford University, United Kingdom and Stanford University, United States of America.

The remaining authors declare that the research was conducted in the absence of any commercial or financial relationships that could be construed as a potential conflict of interest.
